# Natural desiccated thyroid for the treatment of hypothyroidism?

**DOI:** 10.3389/fendo.2023.1309159

**Published:** 2024-01-08

**Authors:** Adrian H. Heald, Peter Taylor, Lakdasa Premawardhana, Mike Stedman, Colin Dayan

**Affiliations:** ^1^ The School of Medicine and Manchester Academic Health Sciences Centre, University of Manchester, Manchester, United Kingdom; ^2^ Department of Endocrinology and Diabetes, Salford Royal Hospital, Salford, United Kingdom; ^3^ Thyroid Research Group, Division of Infection and Immunity, Cardiff University School of Medicine, Cardiff, United Kingdom; ^4^ Res Consortium, Andover, United Kingdom

**Keywords:** hypothyroidism, treatment unresponsive, NDT, liothyronine, cost

## Abstract

Primary hypothyroidism affects about 3% of the general population in Europe. Early treatments in the late 19^th^ Century involved subcutaneous as well as oral administration of thyroid extract. Until the early 1970s, the majority of people across the world with hypothyroidism were treated with natural desiccated thyroid (NDT) (derived from pig thyroid glands) in various formulations, with the majority of people since then being treated with levothyroxine (L-thyroxine). There is emerging evidence that may account for the efficacy of liothyronine (NDT contains a mixture of levothyroxine and liothyronine) in people who are symptomatically unresponsive to levothyroxine. While this is a highly selected group of people, the severity and chronicity of their symptoms and the fact that many patients have found their symptoms to be alleviated, can be viewed as valid evidence for the potential benefit of NDT when given after careful consideration of other differential diagnoses and other treatment options.

## Introduction

Primary hypothyroidism affects about 3% of the general population in Europe (1). Early treatments in the late 19^th^ Century involved subcutaneous as well as oral administration of thyroid extract (2, 3). Until the early 1970s, the majority of people with this condition were treated with natural desiccated thyroid (NDT) (derived from pig thyroid glands) in various formulations (4), with the majority of people since then being treated with levothyroxine (L-thyroxine).

It is reported that up to 10% of treated diagnosed hypothyroid individuals report impaired quality of life, despite achieving serum free thyroxine and thyroid stimulating hormone (TSH) levels within the laboratory reference range and ideally in the ideally in the lower half of the reference range (5). A proportion of people with hypothyroidism who are seemingly treatment resistant as described, are prescribed liothyronine (L-tri-iodothyronine), usually in addition to levothyroxine and occasionally as monotherapy but some do prefer NDT (6).

From the early 1890s through the mid-1970s, desiccated thyroid was the preferred form of therapy for hypothyroidism. In 1965, approximately 4 of every 5 prescriptions for thyroid hormone in the USA were for natural thyroid preparations (4). Concerns about inconsistencies in the potency of these tablets arose after the discovery that some contained anywhere from double to no detectable metabolic activity (7). It was not until 1985 that the revision of the U.S. Pharmacopeia standard from iodine content to L-liothyronine Tt3)/L-thyroxine (T4) content resulted in stable potency after earlier concerns (8), but by then the move to levothyroxine was nearly complete in many countries so that levothyroxine largely replaced NDT. With its more favorable pharmacokinetics allowing for once daily dosing and clinical trial evidence (9), levothyroxine monotherapy has prevailed as the treatment of choice for primary hypothyroidism.

In some cases, seemingly levothyroxine unresponsive individuals with hypothyroidism, are prescribed a Natural Desiccated Thyroid (NDT) preparation such as Armour Thyroid**^®^** (4) or ERFA Thyroid**^®^**. Other preparations are NatureThroid, WP Thyroid and NP Thyroid (10). They contain a mixture of levothyroxine and liothyronine in a fixed ratio (although this ratio does vary slightly between batches). There is a bovine thyroid derived NDT preparation (11) which can be taken by individuals who for religious or cultural reasons do not eat pork. The NDT preparations available at the time of writing are given in **Table 1**.

**Table 1:** The NDT preparations currently available for prescription in Europe.

Summary of Dessicated Thyroid extracts			
Name	T3 dose	T4 dose	Availability
Natural thyroid (65mg grain)	9mcg	38 mcg	USA
Westhyroid pure (65mg grain)	9mcg	38mcg	USA
NP thyroid (60mg grain)	9mcg	38mcg	USA
Thyroid erfa (60mg grain)	8mcg	35mcg	Europe/Canada
Armour thyroid (60mg grain)	9mcg	38mcg	USA/Europe

Over the last four decades, only a small proportion of clinicians in the United Kingdom in primary care and in specialist endocrinology clinics have prescribed NDT (12). However it remains an agent that is prescribed in the National Health Service (NHS) in England. We previously reported that 436 individuals in 2018/2019. were prescribed NDT in England by their general practitioners (GPs) across 382 general practices (13). In 2022 an estimated 337 individuals were prescribed NDT in general practice in England. However this does not take account of hospital or private prescriptions. In the United States of America (USA), NDT prescribing is much more prevalent by at least a factor of 10 compared with the UK.

The UK National Institute for Health and Care Excellence (NICE), in its clinical guideline on thyroid disease (14), recommended that further research should be undertaken on the clinical and cost effectiveness of liothyronine/levothyroxine combination therapy compared with levothyroxine alone for people with hypothyroidism whose symptoms have not responded sufficiently to levothyroxine alone. In the absence of such evidence, clinicians have had to take a pragmatic approach in relation to the management of patients who report continuing symptoms, in spite of apparent adequate thyroid hormone replacement, with some prescribing NDT as a less costly alternative to liothyronine (13, 15). The average Cost of NDT in 2016 was £207/prescription with growth by 220% to £440 in 2022 still below liothyronine but up on 2016 levels, while total annual prescriptions fell by 44% from 4,257 in 2016 to 2,384 in 2022. However this does not take account of hospital or private prescriptions.

**Figure 1:**
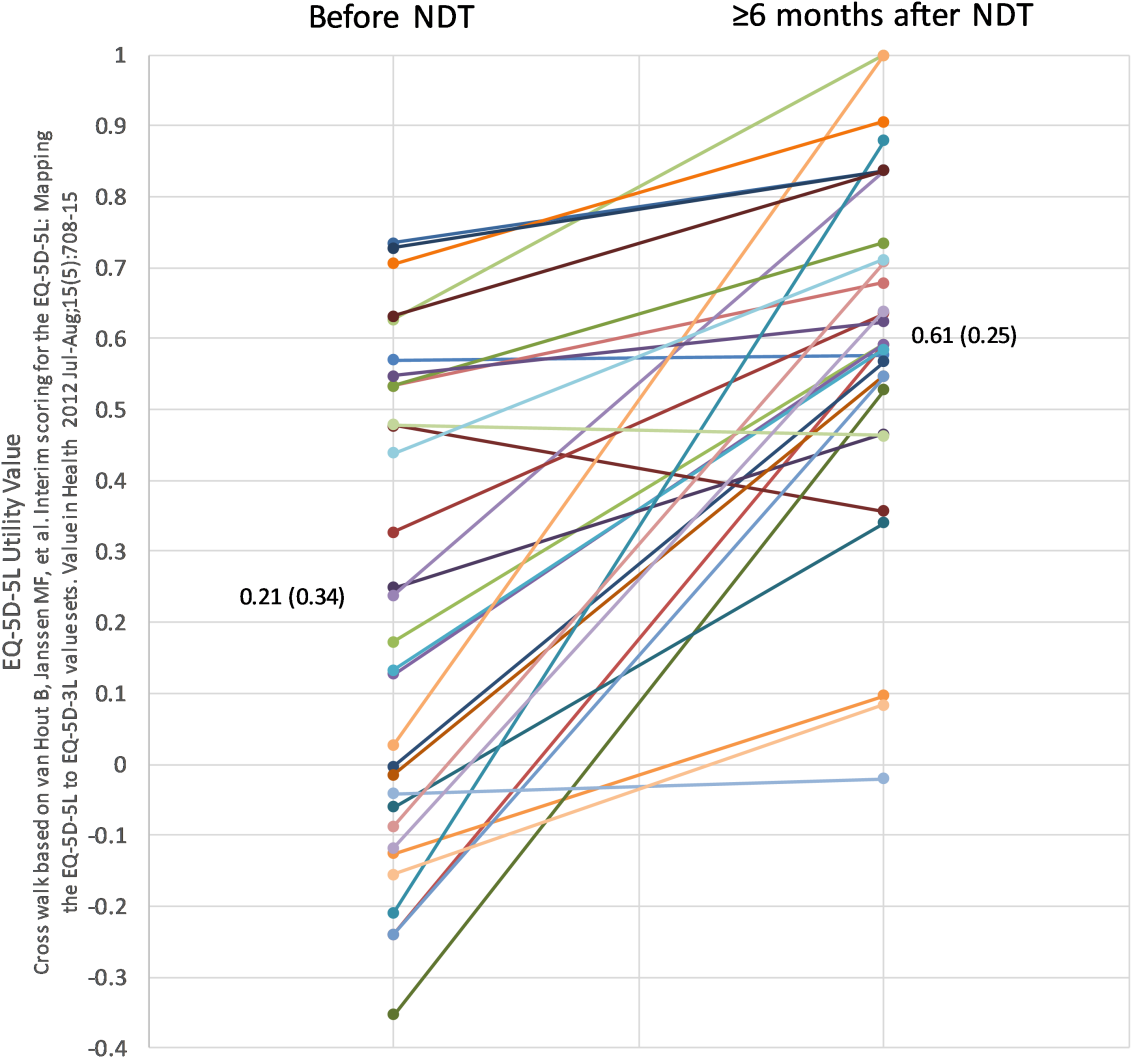
Individual changes in EQ-5D utility scores in relation to NDT treatment.

In our recent case series (16) it was reported that significant benefit was experienced by people who by nature of their lack or response to levothyroxine were treated with NDT. The authors noted that the majority of patients found these symptoms to be alleviated and suggested that this could be viewed as robust evidence for the potential benefit of NDT. Notably a significant associated benefit, as measured both by EQ-5D-5L utility scores and ThyPRO scores was seen in people on NDT, as measured both by the EQ-5D-5L and ThyPRO ratings (**Figure 1**).

Individual descriptions of the response to NDT in relation to improvement in quality of life and reduction in symptoms were as follows in relation to the lived narrative.

As a result of NDT being around for so long it did not ever need to go through the licensing process in North America – it was classed as a ‘grandfathered drug’ (6). The Food and Drug Administration (FDA) allows some unapproved prescription drugs to be lawfully marketed if they meet the criteria of ‘generally recognized as safe and effective’ (GRASE) or grandfathered (17). Grandfathered drugs are those that were already marketed prior to 1938 and were exempt from changes to their labeling or safety studies under the Federal Food Drug and Cosmetics Act (FFDCA) (17). These drugs have not been formally approved by the FDA. NDT is prescribed as part of usual care in both general practice and specialist settings in many countries.

Current guidelines do not endorse the use of NDT but many people across the world do take it and report great benefit. It should be pointed out that thyrotoxic symptoms and some cases of thyroid storm have been reported during the inappropriate use of thyroid hormone extracts including NDT (18). In the United Kingdom and Eire NDT must be prescribed by a health care practitioner and are not obtainable over the counter. In other countries NDT can be bought ‘over the counter’ in person or on-line.

There is emerging evidence that may account for the efficacy of liothyronine (NDT contains a mixture of levothyroxine and liothyronine) in people who are symptomatically unresponsive to levothyroxine (19). Free T3 is the endogenous thyroid hormone, converted from Free T4 predominantly by local de-iodination in tissues. Increased free T4 levels, as seen with levothyroxine therapy alone, appear to inhibit local deiodination except in the pituitary, so that levothyroxine monotherapy may result in TSH (20) inhibition while reducing thyroid bioavailabity in other tissues.

Polymorphisms in the genetic coding of the deiodinase-2 (DIO2) enzyme, present in 13% of the population, have the potential to reduce T3 levels in many tissues, including the brain, without affecting serum levels (21). This may represent a pharmacogenetic component in those who are non-responsive to levothyroxine (22).

The body of opinion continues to be divided as to whether any other option than levothyroxine should be pursued in levothyroxine unresponsive individuals, with NDT among these other options available. Two randomized double blind controlled trials (RCTs) have compared NDT with levothyroxine. In the RCT reported by Hoang et al. (19), NDT therapy did not result in a significant improvement in quality of life. However NDT was associated with a degree of weight loss and nearly half (48.6%) of the study participants expressed preference for NDT over levothyroxine. (48.6%) Those who preferred NDT lost 4lb during the treatment, and their subjective symptoms were significantly better while taking DTE as measured by the general health questionnaire-12 (23) and a bespoke thyroid symptom questionnaire (24). In a subsequent study by the same group involving a comparison between levothyroxine, levothyroxine+ liothyronine and NDT, subgroup analyses of the 1/3 most symptomatic patients on levothyroxine revealed strong preference for treatment containing liothyronine (levothyroxine+liothyronine and NDT), which reduced symptomatology and improved performance on several scales (25).

The American Thyroid Association concluded in 2014 that there is a role for long-term outcome clinical trials testing combination therapy or thyroid extracts (26). We accept that there are some who take a different view on the prescribing of NDT to people with levothyroxine unresponsive hypothyroidism, for example by Coutinho et al. in 2018 (27) – they stated that due to the ‘lack of standardization’ in the liothyronine content, the use of ArmourThyroid^®^ should be avoided. However, it should again be pointed out that since 1985 the formulation of clinician prescribed NDT has been considered reliable (8), contrary to what is believed by some to be the case. Nevertheless the precise composition of NDT thyroid does very slightly over time, given that this is a biological preparation.

In countries like the UK, where liothyronine prescription costs run at a premium (although they have reduced in recent years), NDT may be considered as a lower dose alternative to combination treatment with levothyroxine and liothyronine. Nevertheless it should not be prescribed in anyone with a history of diagnosed cardiac dysrhythmia.

Some clinicians take a different view on the matter of alternative treatment of levothyroxine (28). The reduction it symptoms and improvement in quality of life, as evidenced in our study by the change in scores on the ThyPro and EQ5D5L (29, 30) scales provide objective evidence of benefit and although very much a minority player in terms of numbers of prescriptions in the UK (30) and in some other countries, it is still seen by some clinicians as an agent that be of benefit in treating people with ongoing symptoms of hypothyroidism in spite of levothyroxine treatment. Furthermore individual patient preference in relation to medication that most people with hypothyroidism have to take lifelong should be considered when making prescribing choices.

For initiation of NDT, the therapy is usually instituted using low doses, with increments which depend on the cardiovascular status of the patient. The usual starting dose is 30 mg of eg Armour Thyroid (31) with increments of 15 mg every 2 to 3 weeks. A lower starting dosage, 15 mg/day, is recommended in patients with long-standing myxedema, particularly if cardiovascular impairment is suspected, in which case extreme caution is recommended. The appearance of angina is an indication for a reduction in dosage. Most patients require 60 to 120 mg/day. Failure to respond to doses of 180 mg suggests lack of concordance or malabsorption. Maintenance dosages 60 to 120 mg/day usually result in normal serum T4 and T3 levels. Adequate therapy usually results in normal TSH and T4 levels after 2 to 3 weeks of therapy.

Individual descriptions of the response to NDT in relation to improvement in quality of life and reduction in symptoms (**Table 2**) have validity in a clinical context. The potential beneficial effects of combination treatment with levothyroxine and liothyronine (with an optimum ratio of LT4 to LT3) cannot be compared to treatment with NDT in hypothyroid patients; the latter containing a slightly different proportion of LT3 and LT4 vs the physiological ratio. We need large well-designed RCTs to identify which individuals could benefit from NDT compared to LT4 monotherapy.

**Table 2:** Description of patient experience on going on NDT Thyroid.

Activities	Energy / Fatigue	Sleep / Psychological Symptoms	Physical Health	For those who stopped the NDT
I can go for a walk every day now, still 25% to go Life became brighter. I found it easier to make tough decisions. My coping mechanisms were much improved. I feel now, as I did when I was young. Since going on NDT I have more motivation. I can get up in the morning more easily. I can think more clearly. I can go to college. There is less brain fog and I am able to concentrate on my assignments and project work.	I have a little more energy, I am less puffy minded and more alert. Also, I sleep better. NDT generally made me feel better then when I was on Thyroxine. It has done great things for me. Before taking NDT, I was on the couch all the time. Prior to starting NDT I was treated with Levothyroxine for years for my hypothyroidism. No matter what dosage I was on, I never felt better and my symptoms never improved. I had severe fatigue, dry skin, hair loss, no motivation, severe brain fog, regular digestive issues and a poor quality of life. Since going on NDT my energy levels have improved and my symptoms have lessened. I sleep better, my skin is better and the brain fog I used to experience has lifted considerably from what it used to be. I feel like I can participate more in life now than before.	I look and feel different. Since going on NDT I can sleep better, I am more focussed, I have no anxiety and tension has gone. I feel more and more myself. There is a sweet spot between what is too little and too much NDT. With NDT life became a lot calmer. I found it easier to make tough decisions. My coping mechanisms improved. I started to feel as good as I did when I was younger. On NDT I feel more energetic and less anxious. I feel more able to do things and this has a very positive effect on my life day to day. NDT has completely changed my life. Before going on it I could not see any point in life. Life was miserable. NDT has completely saved my life. I lost a lot of years sleeping and feeling horrendous from my twenties. Within a week of starting NDT I emerged from the fog. On the 120mg dose I feel reasonably well and more sparky. I did notice subtle improvements in other areas I wasn’t expecting like memory and over time less instances of feeling lower in mood. On Levothyroxine, I was depressed and suicidal, I had trouble getting out of bed as my life seemed pointless and everything felt so difficult, my joints hurt so I couldn't exercise or walk, I was bloated, my digestion was horrendous, my periods had me faint in pain, I didn't want to live as it was seriously miserable. NDT made so much of a difference, I could work again, I could literally live, my career picked up, the depression went to sometimes feeling a bit down but nothing like the black hole that I felt sucked me in before. I am married, before NDT my husband felt more like a care giver, after NDT we went back to being partners and me not being that shell that doesn't take part in anything and cries most of the time, but actually the woman he did marry, who shared his life and his interests. It was life changing, I couldn't believe how much	It is difficult to answer these questions because of the other health conditions that `I have. On the good side the eczema has cleared up. I’m not losing my hair now. I’m not the bloated tired pathetic lump that I was before NDT. I would not trade my life now for what it was then. The stuff has been so beneficial and so good for me. I would not want to live without it. Like night and day. Everything was like having a cloud over me then it went way. Everything is now easy compared to life before NDT. I spent years on T4 – I felt like I was going mad. I was on Levothyroxine for 12 or 13 years. For the first time in all those years when I started NDT 120mg I felt like a normal person again. The dry skin and constipation are gone. I am sharp in mind and I have more energy. I have less pain. I can do more. I am no longer cold. My body still itches but on Levothyroxine my body was unbearable. Now my body is happy to have NDT inside it.	After some tine on the NDT Thyroid I experienced a “double heart beat” and deprivation of my sleep pattern, in particular an increased difficulty in going off to sleep. There was regular chest pain on exertion and also tachycardia so I had to stop the medication. NDT Thyroid, although linked with a slight improvement in energy level in the initial stages did not relate to any sustained improvement in my energy level nor any decrease in my fatigue.

Nevertheless, while NDT is not perfect in terms of the ratio of liothyronine to levothyroxine it can offer a ‘lifeline’ to people who may for many years have experienced debilitating symptoms of levothyroxine unresponsiveness who are not able to access combination prescribed LT4 and LT3.

## Conclusion

Significant benefit is experienced by people who by nature of their lack or response to levothyroxine have been treated with NDT. While this is a highly selected group of people, the severity and chronicity of their symptoms and the fact that many patients have found their symptoms to be alleviated, can be viewed as valid evidence for the potential benefit of NDT when given after careful consideration of other differential diagnoses and other treatment options.

## Author contributions

AH: Conceptualization, Investigation, Methodology, Resources, Visualization, Writing – original draft, Writing – review & editing. PT: Conceptualization, Investigation, Software, Visualization, Writing – original draft, Writing – review & editing. LP: Conceptualization, Formal Analysis, Investigation, Methodology, Writing – original draft, Writing – review & editing. MS: Validation, Visualization, Writing – original draft, Writing – review & editing. CD: Conceptualization, Validation, Writing – original draft, Writing – review & editing.
